# Effects of Food Enrichment Based on Diverse Feeding Regimes on Growth, Immunity, and Stress Resistance of *Nibea albiflora*

**DOI:** 10.3390/antiox14121446

**Published:** 2025-11-30

**Authors:** Yuhan Ruan, Jipeng Sun, Yuting Zheng, Jiaxing Wang, Dongdong Xu, Tianxiang Gao, Anle Xu, Xiumei Zhang

**Affiliations:** 1 Fisheries College, Zhejiang Ocean University, Zhoushan 316022, China; ruanyuhan0717@163.com (Y.R.); zhengyuting0615@163.com (Y.Z.); gaotianxiang0611@163.com (T.G.); 2 Research Office of Marine Biological Resources Utilization and Development, Zhejiang Marine Development Research Institute, Zhoushan 316021, China; jipengsun@yeah.net (J.S.); 18868005756@163.com (J.W.); 3 Key Lab Mariculture & Enhancement, Zhejiang Marine Fisheries Research Institute, Zhoushan 316021, China; xudong0580@163.com

**Keywords:** food enrichment, *Nibea albiflora*, physiology and histology analysis, functional genes, healthy culturing

## Abstract

Food enrichment represents a novel feeding strategy for aquaculture. In the current study, juvenile *Nibea albiflora* (average weight 29.65 ± 0.13 g) were used and three feeding regimes (A—commercial diet; B—a diet comprising 90% commercial feed and 10% ice-fresh *Palaemon gravieri*; C—a diet consisting of 90% commercial diet, 5% ice-fresh *Palaemon gravieri* and 5% live *Perinereis nuntia*; named control group, Group 1, and Group 2) with comparable nutrient compositions: were designed to establish the food enrichment model and explore the effects of such feeding strategies on the fish. The cultivation period was 60 days, and the physiological, pathological, and RNA-seq analyses were performed to evaluate the effects. The results showed that the food enrichment feeding strategy significantly enhanced fish growth performance, immunity, and stress resistance without increasing the unit production cost (UPC). Furthermore, the tri-combined food feeding (C) was better than the two-combined food feeding (B). Liver transcriptomic analysis revealed that, in the comparison between the control group and Group 1, the up-regulated genes (*alox15b*, *gng7*, *hif1a*, *ppara,* and *pla2g*) and down-regulated genes (*ins*, *gck*, *il4i1*) influenced fish physiology and further improved growth. Similar to the comparison between the control group and Group 2, the major functional genes included *ugt*, *nlrp3*, *mx1*, *col1a*, *gst* (up-regulated), and *map2k1*, *myc*, *mmp9*, *wnt7*, *socs3* (down-regulated) that participated in regulating the body growth, immunity, and stress resistance. The up-regulated genes (*ins*, *mhc2*, *foxo3*, *ppara*, and *mx1*) alongside the down-regulated genes (*egfr*, *fos*, *cyc*, *myc,* and *mmp9*) probably contributed to the enhanced efficacy of the tri-combined food feeding compared to the two-combined food feeding. In summary, this study demonstrates the beneficial effects of such a food enrichment model on the fish and provides empirical evidence supporting the implementation of the feeding strategies in the healthy culturing of the fish.

## 1. Introduction

Enrichment is a crucial strategy for enhancing aquatic animal survival [1], health [2], welfare [3], natural behavior [4], etc. It is defined as an increase in environmental complexity and can be divided into non-food-based items and food-based items, including physical, social, sensory, occupational, and food enrichment [5]. Numerous studies have documented the positive impacts of the first four enrichment categories in fisheries [6,7,8,9,10], while few have focused on food enrichment.

Food enrichment is a notable feeding strategy recognized for its potential to enhance the welfare of captive animals. It can be achieved by modifying the form or type of food, adjusting the feeding times or frequencies, diversifying feeding methods, or tailoring feeding strategies based on animals’ dietary characteristics [11]. The earliest report concerning the effects of food enrichment on animals can be dated back to 1956 [12], and the research demonstrated that under conditions of extreme starvation, rats subjected to prolonged confinement predominantly sought food along the long-straight pathway instead of the short-food adequacy pathway because it demanded more exploration. The observed phenomenon revealed that the captive living style resulted in stereotypic behaviors in animals and potentially diminished their natural survivability, inspiring efforts to enhance the well-being of captive animals. By now, the efficacy of food enrichment feeding regimes on animals has been predominantly focused on studies in mammals [13,14], reptiles [15,16], amphibians [17] and birds [18,19], and its advantages include meeting animals’ diverse nutritional requirement [20], improving the immunity [21], training fine action and cognitive ability [22], enhancing memory [23] and stimulating social interaction [24], supporting that food enrichment could play an important role in improving the captive animals’ daily life, maintaining their health, enhancing their ability for seeking food, environment adaptation, and so on. However, the impact of food enrichment on aquatic animals remains insufficiently explored.

In aquaculture, several researchers have investigated the effects of food enrichment. For instance, Kazemi et al. [25] examined the impact of feeding *Oncorhynchus mykiss* with *Artemia nauplii* of varying vitality and found that this approach could improve the species’ growth performance, survival rates, and environmental adaptability. Six diatom species were used to feed *Haliotis kamtschatkana,* and it was found that *Amphora sauna* supplemented with *Cylindrotheca closterium* constituted an optimal dietary feeding strategy for the survival and growth [26]. Li et al. [27] revealed that feeding frequency could affect *Megalobrama amblycephala* immunity and disease resistance. Mohanta et al. [28] found that two feeding methods (restricted feeding and refeeding) could affect the fish growth and production performance. Additionally, feeding patterns with a self-feeding system were also reported to contribute to the regulation of the physiological condition of farmed fish [29]. All these previous studies provided evidence that the food enrichment model plays an important role in aquaculture. However, the construction of such models can vary, and there remain research gaps to be addressed. For many carnivorous fish, their food compositions are diverse and can include formulated feed, small fish, shrimp, polychaetes, or other options. The alternative food sources can be combined together, to build a food enrichment model to assess their effects on fish.

In our current study, *Nibea albiflora*, an important and promising commercial species in China, was selected, and three estimated levels, including the physiology, the pathology, and the transcriptomics, were performed to elucidate the effects of food enrichment based on various feeding regimes on the fish. The study can enrich us the cognition of food enrichment applied in aquaculture, as well as propose a new approach to efficient and healthy aquaculture.

## 2. Materials and Methods

### 2.1. Ethics Statement

This study was conducted following the National Research Council’s guide for the care and use of laboratory animals and was approved by the Animal Welfare and Ethical Committee of Zhejiang Ocean University (Zhejiang, China) (protocol code 2022046 and 8 August 2022 of approval).

### 2.2. Fish and Food Enrichment Construction

Female *N. albiflora* juveniles were selected to avoid sex-related growth differences [30]. The fish were kindly provided by the laboratory of Professor Dongdong Xu at the Zhejiang Marine Fisheries Research Institute. They were acclimated in artificially prepared seawater under the following environmental conditions: temperature 23–25 °C, salinity 25 ± 1 ppt, pH 7.8–8.5 units, ammonium-nitrogen < 0.05 mg/L, and dissolved oxygen (DO) > 6 mg/L. During the acclimation period, the fish were fed with a commercial pelleted diet (Hanbei Co., Ltd., Huzhou, Zhejiang Province, China) twice per day (8:00 and 17:00) for ten days. Fish feces and excess feed were removed, and 25–50% of the water total volume was renewed after 30 min of feeding.

*N. albiflora* has a greater preference for shrimps and polychaetes [31], and in Zhejiang Province, *Palaemon gravieri* and *Perinereis nuntia* are abundant in resources and low in price. Hence, in this study, the commercial diet, the ice-fresh *Palaemon gravieri,* and the live *Perinereis nuntia* were selected to construct a food enrichment model. The corresponding nutritional composition, including basic nutrients [32], fatty acids [33], amino acids [34], vitamins (by the LC-MS method), and main minerals (measured by the ICP), were analyzed. Specially formulated diets were used to ensure comparable nutritional levels across the three feeding regimes. Finally, the three regimes were A (commercial diet), B (a diet comprising 90% commercial feed and 10% ice-fresh *P. gravieri*), and C (a diet consisting of 90% commercial feed, 5% ice-fresh *P. gravieri,* and 5% live *P. nuntia*), and their nutritional composition is shown in Table S1.

### 2.3. Experimental Design

#### 2.3.1. Feeding Trial

A total of 270 healthy and similar-sized fish (average weight: 29.65 ± 0.13 g) were chosen and randomly divided into three groups with three replicates (30 individuals per replicate in 200 L of water), named the control group, Group 1, and Group 2, and fed with A diet, B diet, and C diet for 60 days, respectively. The feeding and water quality management were similar to those in the acclimation period. The study was conducted during the winter season, with temperature increases achieved solely through the use of heating rods. We regulated the feeding amount according to the daily growth coefficient (DGC) as outlined by Li et al. [35]. Briefly, we calculated the theoretical weight of the next day based on DGC and the initial weight, and then controlled the feeding amount at 3–5% of the theoretical weight. Every ten days, we re-weighed the fish to recalibrate the feeding amounts according to the updated initial weight. For fish in Groups 1 and 2, the feeding amount of the biological food was calculated based on their proportion in the feeding amount, relative to their dry weight.

At the end of the trial, all fish were first fasted for 24 h and then anesthetized in eugenol (1:10,000) prior to weighing, blood sampling, and dissection [36]. The number of fish and their total weight in each tank were recorded to calculate the survival rate and weight gain. After that, all fish from each replicate were equally divided into two parts, and one was used for the feeding trial sampling, and the other was for the dehydration stress assay sampling. Eight individuals per replicate were selected to separately monitor the body weight and body length, then used to collect the blood sample from the caudal vasculature with 1 mL syringes, and finally dissected to obtain the liver and foregut for physiological-biochemical indexes. The livers were weighed, and some were stored for transcriptome analysis. Each tissue from each fish was put into a single frozen tube, then snap-frozen in the liquid nitrogen and finally transported to be stored at −80 °C until use. Another 4 fish were used for histology observation of the liver and dorsal muscle.

#### 2.3.2. Dehydration Stress Assay

After the anesthetized fish were resuscitated, six of them from each replicate were separately removed from the water and placed in the air for 3 min (the duration was designed according to our preliminary experiment result without publication), then quickly moved back to the water, recording the fish respiratory rate for 3 min by the counting machines. Prior to this procedure, the respiratory rate of fish from each replicate was also counted. The recording process involved four personnel: three for observation and one for data recording. Upon completion of each group’s assessment, the water was completely renewed for the subsequent group. Another four fish per replicate were again exposed to the air for 3 min to facilitate the collection of blood samples and livers for further analysis.

### 2.4. Monitoring Indicators and Method

#### 2.4.1. Growth Performance

The growth performance was assessed by the parameters below:

Weight gain (WG, %) = (*W_t_* − *W*_0_)/*W*_0_ × 100.

Specific growth rate (SGR, %/d) = (ln*W_t_* − ln*W*_0_)/*t* × 100.

Condition factor (K, g/cm^3^) = *W_b_*/*L*^3^ × 100.

Protein efficiency ratio (PER) = (*W_t_* − *W*_0_)/*W_p_*.

Hepatosomatic index (HSI, %) = (*W_l_*/*W_b_*) × 100.

Survival rate (SR, %) = (*N*_0_ − *N_t_*)/*N*_0_ × 100.

Unit production cost (UPC, ¥/kg) = TFC/TBG.

*W_t_* and *W*_0_ are the final and initial body weight, respectively, *t* is the feeding days. *L*, *W_l,_* and *W_b_* represent individual fish body length, liver weight, and body weight, respectively. *N*_0_ and *N_t_* represent the initial and final number of fish, respectively. TFC represents the total feed cost, and TBG is the total biomass gain. The calculation method of UPC is referred to [37] with minor modification, supported by the market price that the commercial diet cost per kilogram was RMB 14.3, the ice-fresh *P. gravieri* was RMB 14, and the live *P. nuntia* was RMB 40. Here, we did not take the cost of manual management, water, and electricity into account because they were similar among the three groups.

#### 2.4.2. Physiological and Biochemical Indexes

For each replicate, all fish blood samples were stored at 4 °C for about 12 h, then separately centrifuged at 4 °C, 836× *g*, 10 min, mixing the supernatant to obtain the serum samples. The livers and foregut tissues of four fish from each replicate were homogenized with the ice-cold, 8.6 g/kg physiological saline in an ice bath, and then centrifuged at 4 °C, 3000× *g*, 10 min, to obtain the homogenate. For the serum samples and the liver homogenate, the immunity indexes including the acid phosphatase (ACP), alkaline phosphatase (AKP), lysozyme (LZM), complement 3 (C3) and immunoglobulin M (IgM), as well as the antioxidant indexes consisted of the total superoxide dismutase (T-SOD), catalase (CAT), and the total antioxidant capacity (T-AOC) were monitored. The digestion parameters included lipase (LPS), amylase (AMS), and trypsin in the liver and the foregut, as well as the total cholesterol (T-CHO), total protein (TP), triglyceride (TG), and high-density lipoprotein cholesterol (HDL-C) in the serum and liver were assayed. All these parameters were measured with the same methods used by [36].

### 2.5. Histology Observation

The liver and dorsal muscle were used for histological observation. All samples were first soaked in Bouin’s solution once isolated from the body, lasting for more than 24 h but less than a week. The samples were washed with 70% ethyl alcohol six times to remove the residual Bouin’s solution, then soaked through different levels of ethyl alcohol (from 75% to 100%) to finish the dehydration process, followed by the transparency process, the embedding process, the sliced process, and the H&E staining process. Each sample was cut into slices 5–6 μm thick and observed by Leica DMI 6000B and DM500 model microscopes (Leica Microsystems, Wetzlar, Germany).

### 2.6. Transcriptome Analysis

In order to further investigate the effects of this feeding strategy on the fish, the liver was chosen to perform the transcriptome analysis. The liver RNA of each fish was separately extracted using TRIzol reagent (Vazyme, Nanjing, China), and the RNA sample of each replicate was composed of the four fish liver RNAs mixed in equal amounts. Finally, a total of 9 liver RNA samples were obtained. The quality of these samples was identified via 1% (w/v) agarose gel, Nanodrop 1000 spectrophotometer (NanoDrop, Waltham, Massachusetts, USA), and Agilent 2100 (Invitrogen, Carlsbad, CA, USA), and if they were eligible, they were sequenced to generate the raw reads and clean reads. The clean reads were mapped to the reference genome (GCA_014281875.1) by Tophat2 (V2.1.1), then the expression levels of genes were calculated via RSEM software (V1.3.1) and described with fragments per kilobase of exon model per million mapped fragments (FPKM). Finally, the differential expression analysis for the comparisons (the control group vs. Group 1, the control group vs. Group 2, and Group 1 vs. Group 2) was performed by DESeq2 software (V1.26.0) [38]. The significant differential expression level was set as *p*-value < 0.05 and the |log_2_ (fold change)| ≥ 1. The GO and KEGG enrichment analyses of the DEGs were performed in accordance with the methods introduced by Mao et al. [39] briefly mapping the DEGs to the GO database and identifying their functional terms as biological process, cellular component, and molecular function, using the KEGG Orthology (KO) to integrate pathway and genomic information in the KEGG database. The significant enrichment levels were both set at *p*-value < 0.05. All the raw data have been submitted to the SRA database, NCBI (accession number: PRJNA1034775).

### 2.7. qRT-PCR Verification

A total of 18 genes (6 genes from the comparison of the control group vs. Group 1, 6 from the control group vs. Group 2, and the rest from the Group 1 vs. Group 2) were chosen randomly to verify the veracity and reliability of the transcriptomic results by qRT-PCR. These genes met the criteria that the FPKM > 1 and participated in at least one pathway. The primers were designed by the software Primer 5.0 according to their transcripts, and *β-actin* was set as the reference gene. All the primer information is displayed in Table S2. Each gene was tested in three replications, and each replication was repeated twice to obtain the Ct value. The annealing temperature was set according to the primers (Table S2). The relative expression levels of the target genes were calculated according to the 2^−ΔΔCt^ method [40].

### 2.8. Statistical Analysis

The results were presented as mean and standard deviation (mean ± *SD*), and all data were subjected to Statistical Package for Social Science, release 22.0 (SPSS, Chicago, IL, USA) to perform the statistical analysis. The Levene’s test and the Shapiro–Wilk test were first used to assess the homogeneity of variance and normality for data, respectively, then the one-way analysis of variance (ANOVA) was performed, followed by Duncan’s multiple range test if statistically significant differences occurred. The differences were considered significant at *p* < 0.05.

## 3. Results

### 3.1. Growth Performance

The effects of this food enrichment model on fish growth performance are shown in Table 1. After a 60-day feeding period, compared to the control group, both the two-combined food feeding and tri-combined food feeding significantly enhanced the WG, SGR, FER, and SR, while reducing the HSI (*p* < 0.05), without UPC being increased. Additionally, the tri-combined food feeding significantly increased the K. These results indicated that such a feeding strategy improved the fish growth performance.

### 3.2. Physiological and Biochemical Indexes

The activity of digestive enzymes in the liver, foregut, and stomach was assessed, with results shown in Table 2. The AKP was also listed in the table due to being one of the important parameters to reflect the physiological status of these tissues. Food enrichment feeding strategy significantly influenced the LPS and trypsin activity (*p* < 0.05) but did not affect the AMS activity (*p* > 0.05) in the three tissues. Compared to the control group, the LPS and trypsin activities of the three tissues were all significantly increased in the fish from Groups 1 and 2. The fish from Group 2 had the highest AKP activity among all groups in both the liver and stomach.

The variation in the biochemical indexes, including the TG, HDL-C, and T-CHO, was analyzed, and the results (Table 3) demonstrated that both in the serum and liver, the content of TG and T-CHO had similar trends among groups. In fish from Group 2, the TG was higher, and the T-CHO was lower than that of the control group (*p* < 0.05). The serum HDL-C content in the Group 2 fish was the highest, but the parameter in the liver showed no difference among all groups.

The effects of food enrichment on the antioxidant capacity were analyzed, and a comparison between the results of the feeding trial and dehydration stress assay, which correspond to pre- and post-stress conditions, respectively, was performed (Table 4 and Table 5). The feeding trial results indicated that food enrichment significantly affected the T-AOC, SOD, and CAT in both serum and liver tissues (*p* < 0.05). Meanwhile, it exhibited that fish fed with the tri-combined food generated a higher response than the control group fish to tolerate such stimulation. In the dehydration stress assay, post-exposure to air stress, no significant differences were observed in serum parameters across groups, although a general decreasing trend was noted compared to pre-stress values. In the liver after the stress, significant differences were found among the groups for the three parameters. Both the SOD and CAT of fish from Group 1 and Group 2 were higher than those of the control group. CAT was significantly decreased within-groups pre- and post-stress. T-AOC of Group 2 significantly declined, while the other groups exhibited no significant change when compared pre-stress with post-stress within groups.

Such a food enrichment feeding strategy has the potential to modulate serum immunity by influencing the content of TP, C3, IgM, and activity of ACP, AKP, and LZM (Table 6). Similarly, for the liver, it could influence the activity of ACP, AKP, and LZM (Table 7). In serum, as enhanced by the feeding strategy, the TP content, activities of ACP and AKP in fish from Group 2 were significantly increased, and the LZM activity and IgM content were decreased. However, after the air exposure trial, the LZM activity in the fish from Group 2 was immediately increased and higher than that of the fish from the control group. The IgM in the two groups of fish was decreased to a similar level without a significant difference. The TP content and ACP activity in each group were all decreased after the stress, but the parameters in Group 2 fish were still higher than those of the control group. The C3 content of each group was significantly decreased after the stress, but between the control group fish and the Group 2 fish, before or after the stress, the parameter was at a similar level. In the liver, the activities of ACP, AKP, and LZM in fish of Group 1 and Group 2 were significantly higher than those of the control group fish. After the stress, these parameters in each group almost displayed a declining trend, but no significant difference occurred between the control group and Group 2. No significant difference existed among the three groups according to the values of C3 and IgM content, while the values were decreased due to the air exposure stress, and Group 2 showed more obvious decreases than the control group.

### 3.3. Respiratory Rate Statistics for the Dehydration Stress Assay

**Figure 1:**
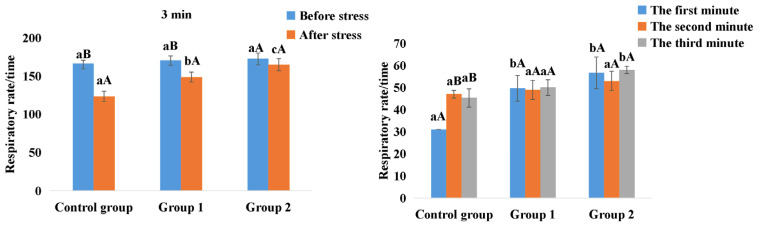
The respiratory rate statistics of the three groups of fish during the 3 min. Values in the same color columns among all groups with different superscript lowercase letters indicate significant differences (*p* < 0.05); meanwhile, values in different color columns within the same group with different superscript capital letters indicate significant differences (*p* < 0.05).

The respiratory rate during the 3-minute period was recorded when the fish was returned from the air back to the water, and the results are shown in Figure 1. Within 3 min, no significant difference was found among all groups before the stress for the total respiratory rate. However, after stress, the index of the control group and Group 1 both decreased, whereas that of Group 2 remained stable. Within each minute of the 3 min, for Group 2, the respiratory rate was always at a stable level, while the control group showed fluctuations, and at the first and third minutes, the index of the control group was lower than that of Group 2.

### 3.4. Histology Observation

**Figure 2:**
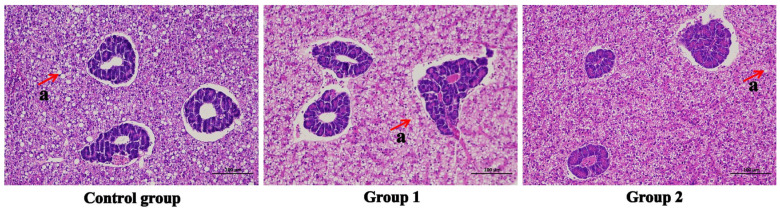
The histology of the livers (magnification, 10 × 20, H&E staining), and the a signed by red arrows represented the fat vacuole.

**Figure 3:**
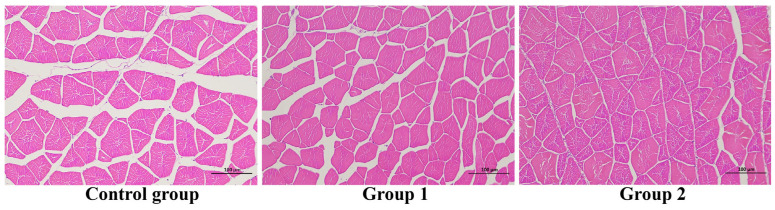
The histology of the dorsal muscle (magnification, 10 × 20, H&E staining).

The histology observation of the liver and dorsal muscle was carried out, and the results were displayed in Figure 2 and Figure 3. Compared to the other groups, the fat vacuole (displayed by the red arrow) was obviously observed in the control group, indicating that food enrichment could make the fish use fat properly. A food enrichment feeding strategy could influence the density of the muscle fascicle.

### 3.5. Transcriptomic Analysis

#### 3.5.1. Analysis of Differentially Expressed Genes

**Figure 4:**
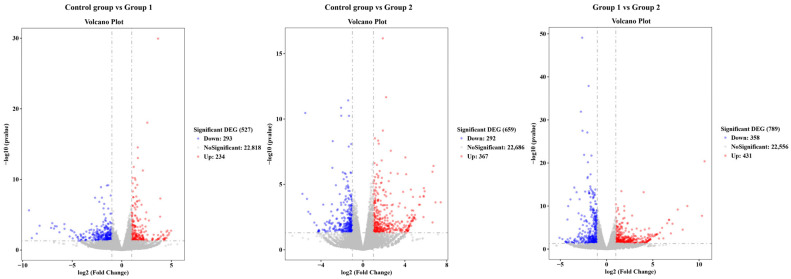
Number of differentially expressed genes and their up- and down-regulated information in the three comparison cases.

The liver was chosen for the transcriptomic analysis so as to further evaluate the effect of the food enrichment on the fish. The pairwise comparisons among all groups were performed. For the comparison of control group vs. Group 1, a total of 527 differentially expressed genes (DEGs) with 293 down-regulated genes and 234 up-regulated genes were found. Similarly, for the comparisons of the control group vs. Group 2 and Group 1 vs. Group 2, the numbers of the DEGs were 659 and 789, respectively, and the corresponding down/up-regulated genes were 292/367 and 358/431 (Figure 4).

#### 3.5.2. GO Enrichment Analysis of Differentially Expressed Genes

The GO enrichment analyses of the DEGs for the three comparisons are shown in Figures S1–S3. Three categories, including cellular component (CC), molecular function (MF), and biological process (BP), were enriched. For the control group vs. Group 1, the DEGs could be enriched into 3883 terms (412 terms in CC, 559 terms in MF, and 2912 terms in BP), and for CC (Figure S1), the DEGs mainly influenced the cellular structure of endoplasmic reticulum (GO:0005783, GO:0005789, GO:0042175, and GO:0044432). Similarly, for MF and BP (Figure S1), the DEGs mainly participate in adjusting the activity of oxidoreductase (GO:0016491, GO:0016705, GO:0016616, etc.) and the metabolism of lipid and nucleotide (GO:0008610, GO:0016125, GO:0006066, etc.), respectively. For the control group vs. Group 2, the DEGs could be enriched into 4507 terms (435 terms in CC, 680 terms in MF and 3392 terms in BP), and for CC (Figure S2), these genes also mainly affected the cellular structure of endoplasmic reticulum, for MF and BP, the genes mostly influenced the activity of some ligase and peptidase (GO:0004175, GO:0070011, GO:0008233, etc.) and were involved in the body immune response (GO:0002440, GO:0016064, GO:0019724, etc.), respectively. For the Group 1 vs. Group 2, 5115 terms (442 terms in CC, 737 terms in MF and 3936 terms in BP) were enriched and for CC, MF, and BP (Figure S3), the DEGs could mainly be enriched in terms related to the cellular structure of mitochondrion (GO:0044429, GO:0005739, GO:0005740, etc.), the proteinase activity (GO:0033764, GO:0016229, GO:0015171, etc.) and transmembrane transport and metabolic of some hormone (GO:1903825, GO:0044281, GO:0051186, etc.), respectively.

#### 3.5.3. KEGG Enrichment Analysis of Differentially Expressed Genes

For the comparison of the control group vs. Group 1, the up-regulated DEGs could be enriched into 132 pathways, and 17 of them were significantly enriched (*p* < 0.05). The major enriched pathways included Arachidonic acid metabolism, Steroid hormone biosynthesis, Pathway in cancer, etc (Figure S4a), and the major functional genes included the *alox15b*, *gng7*, *hif1a*, *ppara,* and *pla2g*. The down-regulated DEGs could be enriched into 145 pathways, and 16 of them were significantly enriched (Figure S4b). The pathways of sugar and amino acid metabolism were significantly enriched, and the functional genes were *ins*, *gck,* and *il4i1.* For the comparison of the control group vs. Group 2, the up-regulated DEGs were enriched into 159 pathways, and 16 of them, such as Drug metabolism-cytochrome P450, Protein digestion and absorption, COVID-19, etc., were significantly enriched, and the genes *ugt*, *nlrp3*, *mx1*, *col1a,* and *gst* were found important in response to the experimental treatment (Figure S5a). Similarly, for the down-regulated DEGs, they could be enriched into 208 pathways, especially the disease and immune response pathways (like Arrhythmogenic right ventricular cardiomyopathy, Hypertrophic cardiomyopathy, Dilated cardiomyopathy, TNF signaling pathway) (Figure S5b), the functional genes included *map2k1*, *myc*, *mmp9*, *wnt7,* and *socs3*. For the comparison of Group 1 vs. Group 2, the up-regulated DEGs could be significantly enriched into 28 pathways, including the metabolism-related (like Protein digestion and absorption, Fatty acid biosynthesis, Fat digestion and absorption) and immune-related pathways (like Ferroptosis, AMPK signaling pathway, Intestinal immune network for IgA production) (Figure S6a), and the major genes included *ins*, *mhc2*, *foxo3*, *ppara* and *mx1*. The down-regulated DEGs could be significantly enriched into 27 pathways, and most of them were the disease-related pathways (like Acute myeloid leukemia, Viral myocarditis, Systemic lupus erythematosus) (Figure S6b). The functional genes consisted of *egfr*, *fos*, *cyc*, *myc,* and *mmp9*.

The trend analyses of the DEGs among the three groups were also executed, and the DEGs mainly functioned in metabolism-related (Arachidonic acid metabolism), disease-related (Arrhythmogenic right ventricular cardiomyopathy), and immune-related (Notch signaling pathway) pathways (Figure S7). The major functional genes included the *itga6*, *cacna2d2* (down-regulated), *nlrp3*, *ccnb1*, *alox15b* (up-regulated).

#### 3.5.4. Differentially Expressed Genes Verification by qRT-PCR

A total of 18 DEGs (six genes consisting of three up-regulated and three down-regulated from each comparison case) were selected to verify the result of RNA sequencing and analyses. All the genes’ relative expression levels demonstrated a similar trend with the RNA-seq (*R*^2^ = 0.9841, Figure S8), indicating that the results provided by the RNA-seq are generally reliable.

## 4. Discussion

While food enrichment is a long-standing practice in zoo management, crucial for enhancing animal welfare and encouraging natural behaviors, it is a comparatively new concept in aquaculture [14,15,17,19]. Given this context, the potential application of such a feeding strategy in aquaculture merits significant research attention. In our current study, as detailed in Table S1, the primary differences among the three diets pertain to protein content, which ranged from 47.06% to 50.10%, and vitamin E concentration, which ranged from 4.54 mg/kg to 11.52 mg/kg. However, previous research has indicated that dietary protein levels between 46.52% and 49.84% or vitamin E levels between 4.0 mg/kg and 15.4 mg/kg do not significantly affect growth performance or other physiological functions in *N. albiflora* [41,42]. This suggests that the variations in protein content and vitamin E concentration among the three groups are unlikely to interfere with the experimental outcomes. The results demonstrate that, following the food enrichment, the fish WG, SGR, FER, and K were significantly increased, and the density of muscle fascicle was also increased, indicating that such a feeding strategy could improve the body growth [43]. The SR in fish from Group 1 and Group 2 was higher than that of the control group, which suggested that food enrichment could improve survival. High HSI was reported as a signal that reflects fatty liver disease [44,45,46], similar to our study, combined with the histology observation result that more fat vacuoles were found in fish from the control group, the HSI in fish from Group 1 and Group 2 was lower than the control group, indicating that food enrichment could protect the liver from fat deposition, to decrease the risk of disease. As shown by the parameters, we propose that food enrichment can improve the fish growth performance with no increased production cost, and the tri-combined food feeding seems to be better than the two-combined food feeding.

The effects of food enrichment on digestion were evaluated. Consistent with other carnivorous fishes [47,48], *N. albiflora* preferentially utilized protein and lipid over carbohydrate; therefore, the AMS activity among groups in the three digestive organs was not significantly influenced. The LPS activity in the liver was significantly increased when fed using the food enrichment model, indicating that the feeding strategy could promote the utilization of lipids, as corroborated by the results of histology observation and HSI. The increase in the LPS and trypsin activity in each tissue of the Group 2 demonstrated that the fish showed high utilization efficiency for the nutrients, while the high AKP activity contributed to enhancing the absorption of nutrients [49] increased the WG.

The tri-combined food feeding decreased the TG content and increased the HDL-C content. High TG content in the serum probably induces atherosclerosis [50], and the HDL-C can help to transport the TG to the liver to be metabolized [51]. The variation in the two parameters revealed that such a feed strategy could benefit the fish’s health. In the liver, the T-CHO content was gradually increased as the diversity of the food elements; we speculated that the food enrichment probably enhanced the transport of the T-CHO into the liver for metabolism [36]. Meanwhile, it also facilitated T-CHO participation in maintaining the stability of the liver cell membrane, and once homogenized at a high speed (>10,000 rpm), the substance was released from the phospholipid bilayer [52].

According to respiratory rate data from the dehydration stress assay, fish in Group 2 displayed no significant difference before and after the stress exposure, indicating a robust adaptability to stress following the tri-combined food feeding. This adaptability is further corroborated by changes in levels of T-AOC, SOD, and CAT. The increase in the three parameters in the serum and liver signaled that the tri-combined food feeding could enhance the body’s anti-stress capacity [53,54]. Meanwhile, after the stress, the Group 2 fish presented a sensitive response to the stress by consuming the antioxidants at a high amplitude.

The immune-related physiological indexes in the serum and liver were monitored to explore the effects of food enrichment on the immunity. As shown in Table 6, fish fed with the tri-combined food had higher TP content and AKP, ACP activity than that of the control group in the serum. For the LZM, C3, and IgM, although the parameters were not higher than those of the control group, they almost displayed a similar immune level to the control group after the stress. The TP contains many immunoglobulins and complement components [55], and to some extent, the increase in the parameter can reflect that the immunity is promoted [56]. The AKP and ACP were proven to play an important role in enhancing the body’s immunity [57]. The variation in these parameters indicated that the immunity of fish in Group 2 was improved. Meanwhile, according to the variation in the LZM, C3, and IgM, the fish in Group 2 were probably in a silent status in producing the immune substance so as to spare more energy for growth or other physiological demands. The phenomenon was also revealed in the articles [58,59,60]. In the liver (Table 7), both the fish from the Group 1 and Group 2 had higher activities of AKP, ACP and LZM than that of the control group; however, after the stress, these parameters in Group 2 fish were declined to as same level as that of control group fish, indicating that the Group 2 fish would generate a higher response than the control group fish to tolerate such stimulation. Similar changing trends were also observed in the parameters of C3 and IgM, which suggested that such food enrichment could enhance body immunity and stress resistance.

The GO enrichment analysis of the DEGs supported by the comparisons of the control group vs. Group 1 (Group 2) uncovered that these genes could influence the function of endoplasmic reticulum (GO:0005783, GO:0005789, GO:0042175, and GO:0044432), which might provide some evidence that the food enrichment can cause the body to synthesize antioxidants or some proteases. Meanwhile, the comparison of the control group vs. Group 2 revealed that the DEGs could be significantly enriched in immune-related terms (GO:0002440, GO:0016064, GO:0019724), which provided further evidence that the food enrichment influenced the body’s immunity.

According to the KEGG enrichment analysis, for the comparison of the control group vs. Group 1, the up-regulated genes (*alox15b*, *gng7*, *hif1a*, *ppara,* and *pla2g*) and down-regulated genes (*ins*, *gck*, *il4i1*) were found to function in influencing the fish growth, immunity, and antioxidant capacity. The *alox15b* was revealed to regulate the body’s immune and inflammatory response [61,62]. The up-regulation of *gng7* was proven to maintain body health [63,64]. Dourado et al. [65] reported that normoxic group fish had higher expression of *hif1a* than that of the hypoxic group fish. The *ppara* is involved in regulating inflammatory pathways and reducing fat deposition [66,67], and the *pla2g* is a lipid target gene that can influence lipid metabolism [68]. Previous studies demonstrated that *Epinephelus fuscoguttatus* ♀ × *Epinephelus lanceolatus* ♂ under normal conditions would have lower expression of *ins* and *gck* in the liver than that of the unhealthy one [36]. The *il4i1* was found to be a key gene induced by some operation that functioned in the immune response [69]. The differential expression of these genes demonstrated that such a food enrichment could influence the fish physiology, which was to the benefit of the species’ growth. For the comparison of the control group vs. Group 2, the fish immunity and antioxidant capacity were significantly increased, probably related to the upregulation of the genes *nlrp3* [70], *mx1* [71], *col1a* [72], and *gst* [73]. Meanwhile, these genes might help the body to maintain homeostasis so as to influence growth performance [72,74]. Opposite to the genes *myc* [75], *mmp9* [76], *wnt7* [77], *socs3* [78], their down-regulation would benefit fish health. The Group 2 fish displayed a better physiological status than that of the Group 1 fish. This might be owing to the differential expression of the genes *mhc2* [79], *foxo3* [80], *ppara* [66,67], *mx1* [71] (up-regulated), and *egfr* [81], *fos* [82], *myc* [75], *mmp9* [76] (down-regulated) that enhanced body health.

## 5. Conclusions

Food enrichment is a novel feeding strategy for aquaculture. The present study demonstrated that the food enrichment model constructed by various feeding regimes could significantly improve the growth performance, immunity, and stress resistance of the fish. Notably, the tri-combined food feeding (Group 2) outperformed the two-combined food feeding (Group 1). The transcriptomic analysis revealed that for the comparison of the control group vs. Group 1, the up-regulated genes (*alox15b*, *gng7*, *hif1a*, *ppara,* and *pla2g*) alongside down-regulated genes (*ins*, *gck*, *il4i1*) positively influenced fish physiology, thereby benefiting species growth. Similarly, for the comparison of the control group vs. Group 2, the major functional genes included *ugt*, *nlrp3*, *mx1*, *col1a*, *gst* (up-regulated), and *map2k1*, *myc*, *mmp9*, *wnt7*, *socs3* (down-regulated) that participated in regulating body growth, immunity, and stress resistance. The up-regulated genes (*ins*, *mhc2*, *foxo3*, *ppara*, *mx1*) and down-regulated genes (*egfr*, *fos*, *cyc*, *myc,* and *mmp9*) probably made the tri-combined food feeding better than the two-combined food feeding regime.

## Figures and Tables

**Table 1:** Effects of food enrichment based on diverse feeding regimes on the growth performance of *Nibea albiflora*.

Groups	W_0_ (g)	WG (%)	SGR (%/day)	HSI (%)	K (g/cm^3^)	PER (%)	SR (%)	UPC RMB/kg
Control	29.72 ± 0.07	82.30 ± 14.03 ^a^	1.00 ± 0.13 ^a^	1.44 ± 0.02 ^b^	1.23 ± 0.06 ^a^	1.50 ± 0.23 ^a^	86.25 ± 4.78 ^a^	20.63 ± 2.75
Group 1	29.65 ± 0.11	103.73 ± 10.50 ^b^	1.18 ± 0.09 ^b^	1.15 ± 0.06 ^a^	1.26 ± 0.01 ^a^	1.86 ± 0.09 ^b^	95.00 ± 0.00 ^b^	20.96 ± 0.99
Group 2	29.57 ± 0.21	113.51 ± 11.33 ^b^	1.26 ± 0.09 ^b^	1.14 ± 0.04 ^a^	1.38 ± 0.03 ^b^	2.33 ± 0.11 ^c^	98.75 ± 2.50 ^b^	23.24 ± 1.16
*p*-value ^1^	0.204	0.530	0.363	0.169	0.176	0.203	0.013	0.159
*p*-value ^2^	0.013	0.502	0.361	0.025	0.796	0.331	0.072	0.203
*p*-value	0.397	0.015	0.015	0.000	0.006	0.000	0.001	0.141

^1^ *p*-value —the *p*-value of the Levene’s test; ^2^ *p*-value—the *p*-value of the Shapiro–Wilk test. Values in the same columns having the same superscript letters are not significantly different (*p* > 0.05), otherwise, they are significantly different (*p* < 0.05). The same applies below.

**Table 2:** Effects of food enrichment based on diverse feeding regimes on the digestion of *Nibea albiflora*.

Groups	AMS(U/mgprot)			LPS (U/gprot)			Trypsin (U/mgprot)			AKP (U/100 mL)		
	Liver	Foregut	Stomach	Liver	Foregut	Stomach	Liver	Foregut	Stomach	Liver	Foregut	Stomach
Control	1.13 ± 0.07	2.95 ± 0.49	0.27 ± 0.12	2.47 ± 0.15 ^a^	2.35 ± 0.39 ^a^	2.40 ± 0.18 ^a^	3.48 ± 0.62 ^a^	87.70 ± 9.85 ^a^	21.95 ± 4.34 ^a^	50.82 ± 7.42 ^a^	3122.86 ± 294.30	181.42 ± 4.82 ^a^
Group 1	1.11 ± 0.12	2.43 ± 0.21	0.30 ± 0.03	3.41 ± 0.13 ^b^	3.79 ± 0.76 ^b^	2.64 ± 0.30 ^a^	5.15 ± 0.23 ^b^	127.79 ± 11.22 ^b^	25.26 ± 2.65 ^ab^	76.12 ± 2.93 ^b^	3166.05 ± 138.31	173.09 ± 6.47 ^a^
Group 2	1.18 ± 0.07	2.58 ± 0.15	0.17 ± 0.12	3.69 ± 0.27 ^b^	3.63 ± 0.25 ^b^	3.05 ± 0.16 ^b^	6.49 ± 0.92 ^c^	117.01 ± 10.29 ^b^	30.07 ± 1.15 ^b^	95.73 ± 7.94 ^c^	3036.57 ± 134.27	218.25 ± 12.28 ^b^
*p*-value ^1^	0.307	0.183	0.075	0.392	0.209	0.259	0.317	0.963	0.335	0.281	0.132	0.254
*p*-value ^2^	0.425	0.273	0.706	0.207	0.864	0.337	0.968	0.542	0.743	0.837	0.275	0.12
*p*-value ^3^	0.603	0.207	0.306	0.001	0.026	0.032	0.004	0.006	0.044	0.000	0.740	0.001

^1^ *p*-value —the *p*-value of the Levene’s test; ^2^ *p*-value—the *p*-value of the Shapiro–Wilk test; ^3^ *p*-value—the *p*-value of the ANOVA. Values in the same columns having the same superscript letters are not significantly different (*p* > 0.05), otherwise, they are significantly different (*p* < 0.05).

**Table 3:** Effects of food enrichment based on diverse feeding regimes on the serious and hepatic biochemical indexes of *Nibea albiflora*.

Groups	Serum			Liver		
	TG (mmol/L)	HDL-C (mmol/L)	T-CHO (mmol/L)	TG (mmol/gprot)	HDL-C (mmol/gprot)	T-CHO (mmol/gprot)
Control	1.20 ± 0.20 ^b^	2.19 ± 0.13 ^a^	2.21 ± 0.13 ^a^	1.51 ± 0.16 ^b^	0.29 ± 0.06	0.12 ± 0.02 ^a^
Group 1	1.30 ± 0.19 ^b^	2.23 ± 0.17 ^a^	2.57 ± 0.21 ^b^	1.01 ± 0.09 ^a^	0.30 ± 0.08	0.18 ± 0.02 ^b^
Group 2	0.82 ± 0.04 ^a^	2.59 ± 0.16 ^b^	2.88 ± 0.10 ^c^	1.18 ± 0.11 ^a^	0.30 ± 0.08	0.30 ± 0.04 ^c^
*p*-value ^1^	0.208	0.784	0.323	0.425	0.96	0.352
*p*-value ^2^	0.62	0.643	0.552	0.383	0.294	0.518
*p*-value ^3^	0.023	0.036	0.005	0.007	0.992	0.001

^1^ *p*-value —the *p*-value of the Levene’s test; ^2^ *p*-value—the *p*-value of the Shapiro–Wilk test; ^3^ *p*-value—the *p*-value of the ANOVA. Values in the same columns having the same superscript letters are not significantly different (*p* > 0.05), otherwise, they are significantly different (*p* < 0.05).

**Table 4:** Effect of food enrichment based on diverse feeding regimes on antioxidant capacity of the serum.

Groups	T-AOC (mM)		SOD (U/mL)		CAT (U/mL)	
	Feeding Trial	Air Exposure Stress	Feeding Trial	Air Exposure Stress	Feeding Trial	Air Exposure Stress
Control	0.37 ± 0.03 ^aA^	0.46 ± 0.04 ^B^	8.45 ± 0.96 ^bB^	5.84 ± 0.26 ^A^	8.69 ± 0.49 ^ab^	8.22 ± 0.59
Group 1	0.68 ± 0.06 ^bB^	0.51 ± 0.03 ^A^	5.93 ± 0.43 ^a^	5.03 ± 0.38	7.84 ± 0.16 ^aA^	8.87 ± 0.34 ^B^
Group 2	0.71 ± 0.06 ^bB^	0.48 ± 0.01 ^A^	9.29 ± 0.76 ^bB^	5.73 ± 0.38 ^A^	9.58 ± 0.62 ^b^	8.73 ± 0.40
*p*-value ^1^	0.432	0.351	0.49	0.604	0.167	0.377
*p*-value ^2^	0.061	0.902	0.369	0.414	0.437	0.396
*p*-value ^3^	0.000	0.178	0.004	0.057	0.011	0.266

^1^ *p*-value —the *p*-value of the Levene’s test; ^2^ *p*-value—the *p*-value of the Shapiro–Wilk test; ^3^ *p*-value—the *p*-value of the ANOVA. Values in the same columns with different superscript lowercase letters indicate significant differences (*p* < 0.05); meanwhile, values of one parameter in the same row with different superscript capital letters indicate significant differences (*p* < 0.05).

**Table 5:** Effect of food enrichment based on diverse feeding regimes on antioxidant capacity of the liver.

Groups	T-AOC (mmol/gprot)		SOD (U/mgprot)		CAT (U/mgprot)	
	Feeding Trial	Air Exposure Stress	Feeding Trial	Air Exposure Stress	Feeding Trial	Air Exposure Stress
Control	0.10 ± 0.01 ^aA^	0.12 ± 0.01 ^bB^	20.00 ± 5.52 ^a^	15.73 ± 4.22 ^a^	8.352 ± 2.04 ^aB^	3.38 ± 0.85 ^aA^
Group 1	0.12 ± 0.01 ^bA^	0.17 ± 0.01 ^cB^	20.18 ± 3.39 ^aA^	36.35 ± 3.13 ^cB^	15.35 ± 1.84 ^bB^	10.35 ± 0.80 ^bA^
Group 2	0.13 ± 0.01 ^bB^	0.10 ± 0.01 ^aA^	30.37 ± 3.01 ^b^	25.46 ± 1.15 ^b^	23.27 ± 2.99 ^cB^	14.77 ± 2.15 ^cA^
*p*-value ^1^	0.436	0.258	0.294	0.101	0.645	0.08
*p*-value ^2^	0.765	0.254	0.347	0.742	0.926	0.311
*p*-value ^3^	0.028	0.000	0.035	0.001	0.001	0.000

^1^ *p*-value —the *p*-value of the Levene’s test; ^2^ *p*-value—the *p*-value of the Shapiro–Wilk test; ^3^ *p*-value—the *p*-value of the ANOVA. Values in the same columns with different superscript lowercase letters indicate significant differences (*p* < 0.05); meanwhile, values of one parameter in the same row with different superscript capital letters indicate significant differences (*p* < 0.05).

**Table 6:** Effect of food enrichment based on diverse feeding regimes on serum immunity.

Groups	TP (gprot/L)		AKP (U/100 mL)		ACP (U/100 mL)		LZM (U/mL)		C3 (µg/mL)		IgM (µg/mL)	
	Feeding Trial	Air Exposure Stress	Feeding Trial	Air Exposure Stress	Feeding Trial	Air Exposure Stress	Feeding Trial	Air Exposure Stress	Feeding Trial	Air Exposure Stress	Feeding Trial	Air Exposure Stress
Control	21.02 ± 0.65 ^aB^	11.20 ± 0.27 ^aA^	1.69 ± 0.21 ^aA^	2.12 ± 0.15 ^B^	5.45 ± 0.19 ^aB^	3.82 ± 0.08 ^aA^	34.01 ± 2.17 ^b^	30.00 ± 1.88 ^a^	1088.20 ± 22.39 ^bB^	804.13 ± 69.27 ^bA^	139.91 ± 12.37 ^b^	111.74 ± 13.54 ^b^
Group 1	24.40 ± 1.23 ^bB^	13.09 ± 1.14 ^bA^	2.13 ± 0.05 ^b^	2.05 ± 0.11	5.06 ± 0.36 ^a^	4.85 ± 0.22 ^c^	34.27 ± 2.39 ^bB^	26.46 ± 3.08 ^aA^	881.07 ± 40.90 ^aB^	525.26 ± 88.63 ^aA^	120.80 ± 13.27 ^abB^	76.80 ± 2.59 ^aA^
Group 2	29.64 ± 1.63 ^cB^	14.69 ± 0.64 ^cA^	2.42 ± 0.24 ^bB^	1.90 ± 0.08 ^A^	6.81 ± 0.25 ^bB^	4.29 ± 0.18 ^bA^	28.32 ± 1.23 ^aA^	35.21 ± 2.01 ^bB^	1068.10 ± 46.18 ^bB^	780.13 ± 133.49 ^bA^	109.42 ± 8.46 ^a^	102.74 ± 2.88 ^b^
*p*-value ^1^	0.317	0.196	0.106	0.332	0.481	0.250	0.427	0.405	0.386	0.545	0.579	0.043
*p*-value ^2^	0.559	0.343	0.809	0.912	0.263	0.577	0.804	0.871	0.053	0.626	0.053	0.319
*p*-value ^3^	0.000	0.004	0.009	0.128	0.001	0.001	0.018	0.012	0.001	0.027	0.047	0.005

^1^ *p*-value —the *p*-value of the Levene’s test; ^2^ *p*-value—the *p*-value of the Shapiro–Wilk test; ^3^ *p*-value—the *p*-value of the ANOVA. Values in the same columns with different superscript lowercase letters indicate significant differences (*p* < 0.05); meanwhile, values of one parameter in the same row with different superscript capital letters indicate significant differences (*p* < 0.05).

**Table 7:** Effect of food enrichment based on diverse feeding regimes on liver immunity.

Groups	AKP (U/100 mL)		ACP (U/100 mL)		LZM (U/mgprot)		C3 (mg/gprot)		IGM (mg/gprot)	
	Feeding Trial	Air Exposure Stress	Feeding Trial	Air Exposure Stress	Feeding Trial	Air Exposure Stress	Feeding Trial	Air Exposure Stress	Feeding Trial	Air Exposure Stress
Control	50.82 ± 7.42 ^a^	39.88 ± 8.40 ^a^	224.02 ± 39.98 ^a^	193.13 ± 45.25 ^a^	9.84 ± 3.38 ^aB^	3.11 ± 0.60 ^A^	193.55 ± 18.39 ^B^	134.39 ± 11.21 ^aA^	31.08 ± 3.24 ^B^	21.15 ± 1.96 ^bA^
Group 1	76.12 ± 2.93 ^b^	66.69 ± 8.04 ^b^	310.98 ± 12.97 ^b^	330.01 ± 63.49 ^b^	19.92 ± 3.47 ^bB^	4.45 ± 0.84 ^A^	194.21 ± 2.20	184.04 ± 28.05 ^b^	24.87 ± 3.12	20.83 ± 3.32 ^b^
Group 2	95.73 ± 7.94 ^cB^	38.46 ± 5.93 ^aA^	313.64 ± 51.44 ^b^	221.88 ± 42.75 ^a^	17.03 ± 2.29 ^bB^	3.38 ± 0.49 ^A^	198.01 ± 27.41 ^B^	116.54 ± 9.60 ^aA^	30.09 ± 4.84 ^B^	15.45 ± 1.68 ^aA^
*p*-value ^1^	0.281	0.777	0.249	0.621	0.813	0.469	0.055	0.225	0.447	0.337
*p*-value ^2^	0.837	0.267	0.688	0.575	0.955	0.505	0.285	0.205	0.384	0.949
*p*-value ^3^	0.000	0.006	0.047	0.038	0.018	0.099	0.954	0.010	0.182	0.049

^1^ *p*-value —the *p*-value of the Levene’s test; ^2^ *p*-value—the *p*-value of the Shapiro–Wilk test; ^3^ *p*-value—the p-value of the ANOVA. Values in the same columns with different superscript lowercase letters indicate significant differences (*p* < 0.05); meanwhile, values of one parameter in the same row with different superscript capital letters indicate significant differences (*p* < 0.05).

## Data Availability

The original contributions presented in this study are included in the article/Supplementary Material. Further inquiries can be directed to the corresponding authors.

## Supplementary Materials

The following supporting information can be downloaded at: https://www.mdpi.com/article/10.3390/antiox14121446/s1, Table S1: Nutrient composition of the three recipes (dry matter); Table S2: Specific primer sequences for the selected differentially expressed genes of the liver. Figure S1: The GO enrichment result of the differentially expressed genes from the comparison of control group vs. Group 1; Figure S2: The GO enrichment result of the differentially expressed genes from the comparison of control group vs. Group 2; Figure S3: The GO enrichment result of the differentially expressed genes from the comparison of Group 1 vs. Group 2; Figure S4: The KEGG enrichment result of the differentially expressed genes from the comparison of control group vs. Group 1, a represents the top 20 of pathway enrichment among the up-regulated differentially expressed genes (DEGs); b represents the top 20 of pathway enrichment among the down-regulated differentially expressed genes, the same below; Figure S5: The KEGG enrichment result of the differentially expressed genes from the comparison of control group vs. Group 2; Figure S6: The KEGG enrichment result of the differentially expressed genes from the comparison of Group 1 vs. Group 2; Figure S7: The KEGG enrichment result of the trend differentially expressed genes among the three groups and the major functional genes analyzed; Figure S8: Results of qRT-PCR for the selected differentially expressed genes (DEGs) compared to RNA-seq.

## Abbreviations

WG	Weight gain
SGR	Specific growth rate
K	Condition factor
PER	Protein efficiency ratio
HSI	Hepatosomatic index
SR	Survival rate
UPC	Unit production cost
TFC	total feed cost
TBG	total biomass gain
