# Supplementary material for: Effects of Food Enrichment Based on Diverse Feeding Regimes on Growth, Immunity, and Stress Resistance of Nibea albiflora
Source: Antioxidants (Basel). 2025 Nov 30;14(12):1446. doi: 10.3390/antiox14121446 (PMC12729953; doi:10.3390/antiox14121446)
Supplement: Supplementary file 1 [file antioxidants-14-01446-s001.zip › antioxidants-3988319-supplementary.pdf]

Table S1

Nutrient composition of the three feeding regimes (dry matter)

Analysis	Pelleted commercial diet	Ice-fresh *Palaemon gravieri*	Live *Perinereis nuntia*	Control group	Group 1	Group 2
**Basic nutrient content (%)**						
Crude protein	47.06	77.46	60.13	47.06	50.10	49.23
Crude lipid	10.45	5.18	7.45	10.45	9.92	10.03
Crude ash	11.56	10.33	8.20	11.56	11.44	11.33
Total energy（kJ/g）	17.31	20.60	17.57	17.31	17.64	17.49
**Fatty acid content (g/100g)**						
C4:0	0.00	0.00	0.00	0.00	0.00	0.00
C6:0	0.00	0.00	0.00	0.00	0.00	0.00
C8:0	0.00	0.00	0.00	0.00	0.00	0.00
C10:0	0.00	0.00	0.00	0.00	0.00	0.00
C11:0	0.00	0.00	0.00	0.00	0.00	0.00
C12:0	0.00	0.00	0.00	0.00	0.00	0.00
C13:0	0.00	0.00	0.00	0.00	0.00	0.00
C14:0	0.23	0.01	0.02	0.23	0.21	0.21
C14:1	0.00	0.00	0.00	0.00	0.00	0.00
C15:0	0.03	0.02	0.03	0.03	0.03	0.03
C15:1	0.00	0.00	0.00	0.00	0.00	0.00
C16:0	1.74	0.30	1.73	1.74	1.59	1.66
C16:1	0.31	0.03	0.13	0.31	0.28	0.28
C17:0	0.04	0.05	0.07	0.04	0.04	0.04
C17:1	0.00	0.00	0.00	0.00	0.00	0.00
C18:0	0.57	0.15	0.55	0.57	0.53	0.55
C18:1n9t	0.00	0.00	0.00	0.00	0.00	0.00

C18:1n9c	1.67	0.21	0.89	1.67	1.53	1.56
C18:2n6t	0.00	0.00	0.00	0.00	0.00	0.00
C18:2n6c	1.86	0.02	1.24	1.86	1.68	1.74
C20:0	0.04	0.02	0.02	0.04	0.03	0.03
C18:3n6	0.01	0.00	0.00	0.01	0.01	0.01
C18:3n3	0.17	0.00	0.16	0.17	0.16	0.16
C20:1	0.06	0.02	0.31	0.06	0.06	0.07
C21:0	0.01	0.01	0.28	0.01	0.01	0.02
C20:2	0.02	0.02	0.88	**0.02**	**0.02**	**0.06**
C22:0	0.03	0.02	0.01	0.03	0.03	0.03
C20:3n6	0.01	0.00	0.07	0.01	0.01	0.02
C20:3n3	0.01	0.00	0.05	0.01	0.01	0.01
C22:1n9	0.01	0.00	0.03	0.01	0.01	0.01
C20:4n6	0.13	0.08	0.29	0.13	0.13	0.14
C23:0	0.01	0.01	0.01	0.01	0.01	0.01
C22:2	0.00	0.00	0.02	0.00	0.00	0.00
C20:5n3	0.55	0.11	0.43	0.55	0.50	0.52
C24:0	0.04	0.01	0.10	0.04	0.03	0.04
C24:1	0.05	0.01	0.01	0.05	0.05	0.05
C22:6n3	0.77	0.12	0.05	0.77	0.70	0.70
**Amino acid content (g/100g)**						
Asp	3.75	6.02	3.71	3.75	3.98	3.86
Thr	1.60	2.20	1.59	1.60	1.66	1.63
Ser	1.68	2.58	1.65	1.68	1.77	1.73
Glu	6.03	9.01	5.34	6.03	6.33	6.15
Gly	2.31	6.88	1.85	2.31	2.77	2.52

Ala	2.37	3.54	2.74	2.37	2.49	2.45
Cys	0.35	0.41	0.34	0.35	0.36	0.35
Val	1.49	2.19	1.37	1.49	1.56	1.52
Met	0.93	1.75	0.64	0.93	1.01	0.95
IIe	1.17	1.69	1.15	1.17	1.22	1.19
Leu	2.92	4.10	2.61	2.92	3.04	2.96
Tyr	1.21	1.86	1.27	1.21	1.27	1.24
Phe	1.81	2.29	1.43	1.81	1.86	1.81
Lys	2.67	4.34	2.75	2.67	2.84	2.76
His	0.94	1.02	0.79	0.94	0.95	0.93
Arg	2.49	5.19	2.18	2.49	2.76	2.61
Pro	1.90	1.87	1.55	1.90	1.89	1.88
**Vitamin content (μg/kg)**						
vitamin B1	764.71	21.02	2566.35	764.71	690.34	817.61
nicotinic acid	1523.59	3139.63	16380.53	**1523.59**	**1685.19**	**2347.24**
Pyridoaldehyde hydrochloride	10.97	93.72	37.60	10.97	19.24	16.44
hydrochloric acid pyridoxine	4292.47	0.00	0.00	4292.47	3863.22	3863.22
vitamin B6	4303.56	94.80	38.06	4303.56	3882.68	3879.84
nicotinamide	5639.48	0.00	0.00	5639.48	5075.53	5075.53
pantothenic acid	2597.17	906.85	5706.70	2597.17	2428.13	2668.13
folic acid	0.00	0.00	0.00	0.00	0.00	0.00
vitamin B12	0.00	0.00	0.00	0.00	0.00	0.00
vitamin B2	1368.26	205.86	8140.45	1368.26	1252.02	1648.75
vitamin C	0.00	0.00	0.00	0.00	0.00	0.00

vitamin B7	0.00	0.00	0.00	0.00	0.00	0.00
vitamin A	0.00	0.00	0.00	0.00	0.00	0.00
vitamin D2	0.00	0.00	0.00	0.00	0.00	0.00
vitamin D3	0.00	0.00	0.00	0.00	0.00	0.00
vitamin K1	134.92	105.24	160.38	134.92	131.95	134.71
vitamin E	4538.77	45893.25	102763.54	**4538.77**	**8674.21**	**11517.73**
**Main mineral substance (mg/kg)**						
Al	1053.77	181.56	109.08	1053.77	966.55	962.92
Cd	0.55	0.39	0.05	0.55	0.54	0.52
Co	0.80	0.23	2.01	0.80	0.74	0.83
Cr	2.10	1.17	0.33	2.10	2.01	1.97
Cu	7.04	25.96	10.01	7.04	8.93	8.13
Fe	1154.61	176.57	397.67	1154.61	1056.81	1067.86
Mn	90.12	18.39	13.49	90.12	82.95	82.70
Ni	1.56	1.33	1.17	1.56	1.54	1.53
Se	0.54	1.72	0.64	0.54	0.65	0.60
Zn	150.11	35.47	44.25	150.11	138.64	139.08
K	10722.22	14265.47	9274.56	10722.22	11076.55	10827.00
Ca	26333.33	59045.82	1083.56	26333.33	29604.58	26706.47
Na	6411.11	24326.88	7300.28	6411.11	8202.69	7351.36
Mg	3700.00	4959.85	2387.51	3700.00	3825.98	3697.37
P	19444.44	9163.91	5234.16	19444.44	18416.39	18219.90

Table S2

Specific primers sequence for the selected differentially expressed genes of the liver

Gene Symbol		Primer sequence	Tm
β-actin	F：	TTATGAAGGCTATGCCCTGCC	T*
	R：	TGAAGGAGTAGCCACGCTCTGT	
**Control *vs* Group 1**			
faxdc2	F：	TTCTTCGGGACAATGACG	54.26
	R：	GATGCGGTAGCGAGTGAT	
hsd17b3	F：	CAGGGCAGATGGTGAAAG	52.86
	R：	GAAGGGAACAGAGGCAAC	
tat	F：	GGTGAAACTGAGCCAACG	54.7
	R：	AGCAGGGAGACAGAAGACA	
eef1a	F：	TCGTCACCTTCGCTCCTG	56.33
	R：	TGATGACCTGGGCGTTGA	
inhbb.4	F：	AGGAGGGTAACTGGCACA	53.02
	R：	CCACAACGCAATGAACG	
mboat7	F：	CCCGATGAGCAGGTGTA	52.32
	R：	TGGCAAAGGGTGTTGGT	
**Control *vs* Group 2**			
nlrp3	F：	ACTTGGCTTACCAGCATTG	52.77
	R：	TCAGCGGTTTGACTTCC	
minpp1	F：	AGGAAGTGAACCCGTATCTC	52.95
	R：	TGCCGTGTCTTATTATTGC	
ugt2b15	F：	TGGAGGTTTGAGGGTGAA	52.48
	R：	AGGAACGCCGTGGTAAAT	
mid1	F：	CCCTGAGCGGACCTAA	51.73
	R：	GCTTCTTGTTGGGATGTG	
dusp1	F：	AAGCCAGGCGAAGAAAG	53.87
	R：	GGTAAGCACTGCCAAGGT	
mboat7	F：	CCCGATGAGCAGGTGTA	52.32
	R：	TGGCAAAGGGTGTTGGT	
**Group 1 vs Group 2**			
gck	F：	CCTGCTTAACTGGACCAAA	51.89
	R：	AACAATCATTCCCACTTCG	
krt18.5	F：	CCAGGCGGTCATTCAG	52.33
	R：	CCAGGCGGTCATTCAG	
gns	F：	AAAGGCTCAGAAACACGG	52.99
	R：	AACGGCTGGTAGTTGGAT	
pck2	F：	GTGGGTGATGACATTGCG	53.305
	R：	GTGGACATGGCGTAGGG	
eif4g2	F：	AGATTCTGGAAGGCAAAGGT	54.98
	R：	TAGATGGACTGTGGTGAGGG	
ulk2	F：	CGGATGACTTTGTTCTGGTG	54.8
	R：	AGGTGGGCTGTGGTTGC	

F is the forward primer, and R is the reverse primer. T* value is the annealing temperature parameter corresponding to the real time internal parameter.

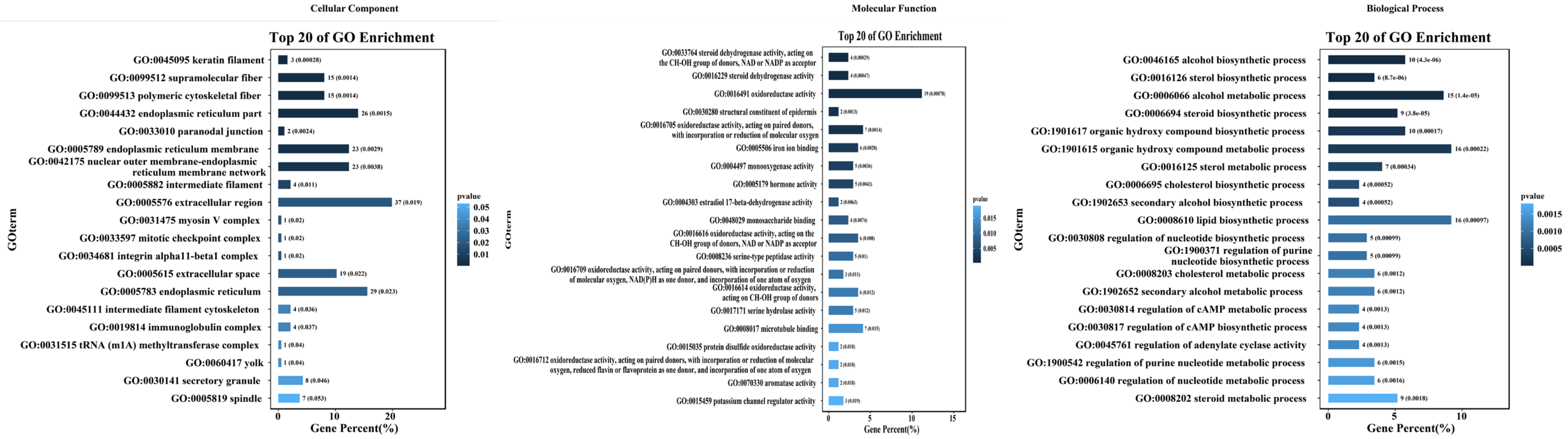

Figure S1 The GO enrichment result of the differentially expressed genes from the comparison of Control group vs Group 1.

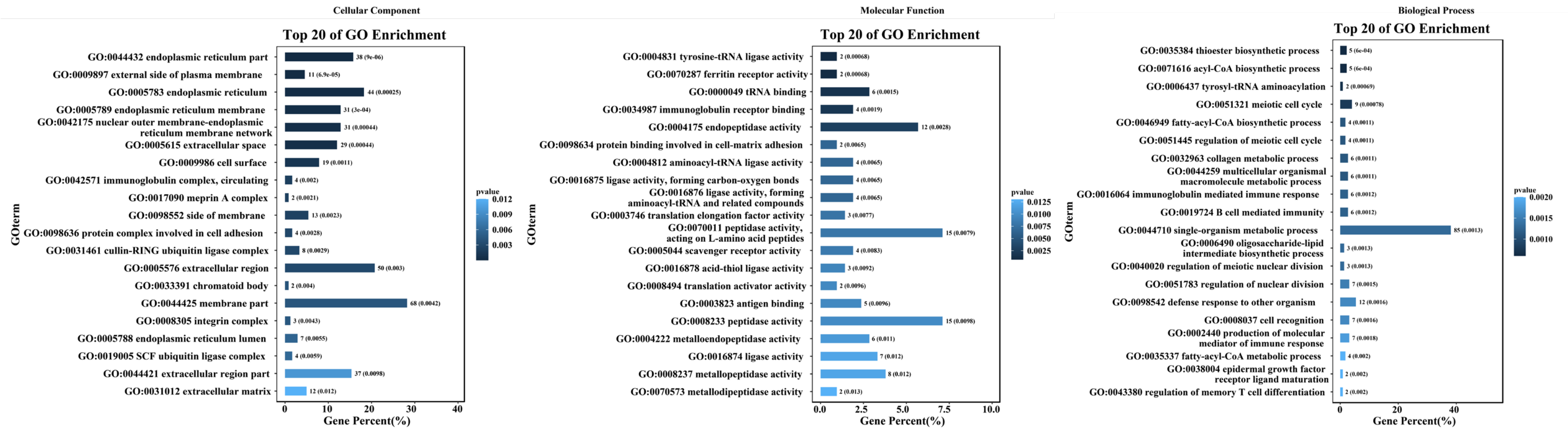

Figure S2 The GO enrichment result of the differentially expressed genes from the comparison of Control group *vs* Group 2.

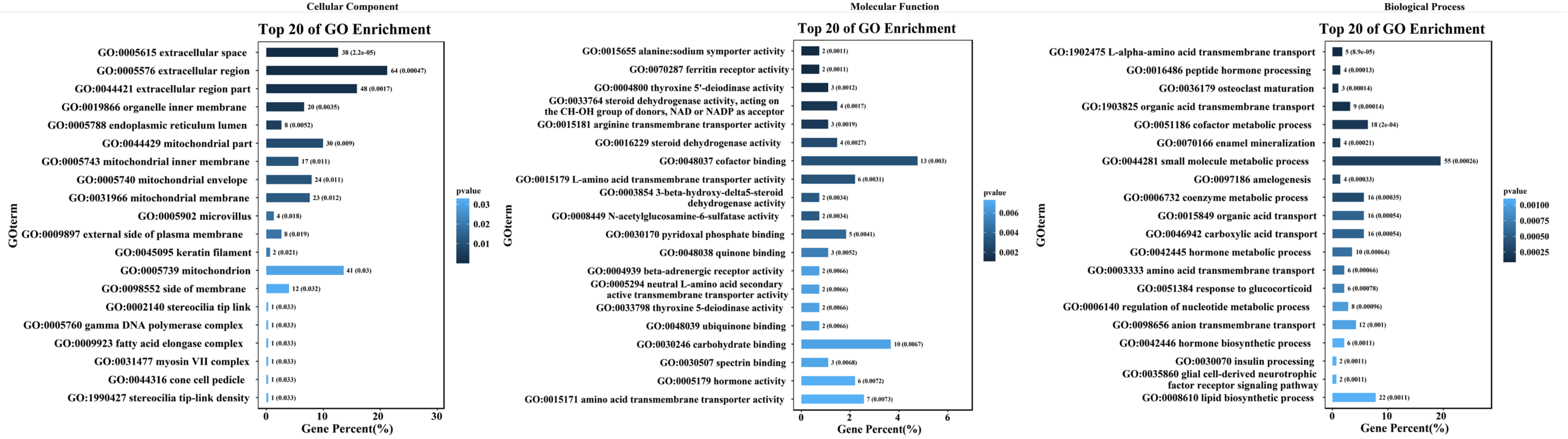

Figure S3 The GO enrichment result of the differentially expressed genes from the comparison of Group 1 *vs* Group 2.

a b

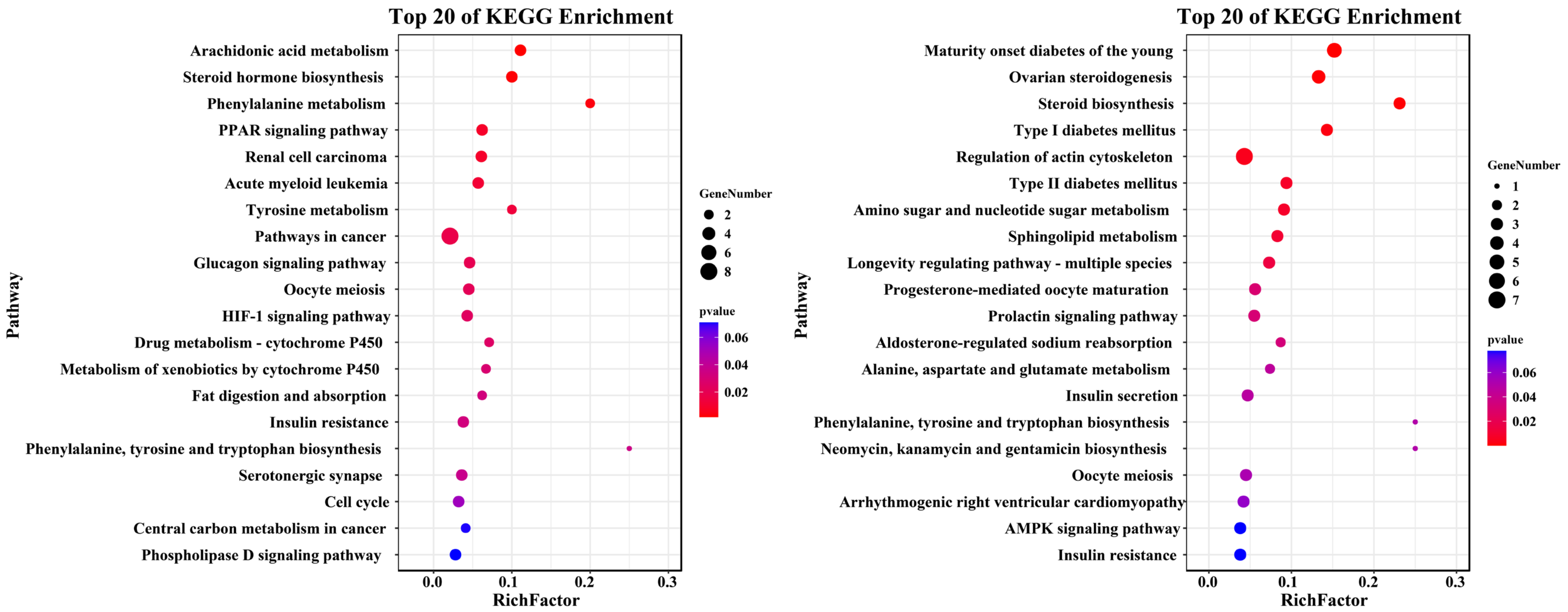

Figure S4 The KEGG enrichment result of the differentially expressed genes from the comparison of Control group vs Group 1, a represents the top 20 of pathway enrichment among the up regulated differentialy expressed genes (DEGs); b represents the top 20 of pathway enrichment among the down regulated differentialy expressed genes, the same below.

a b

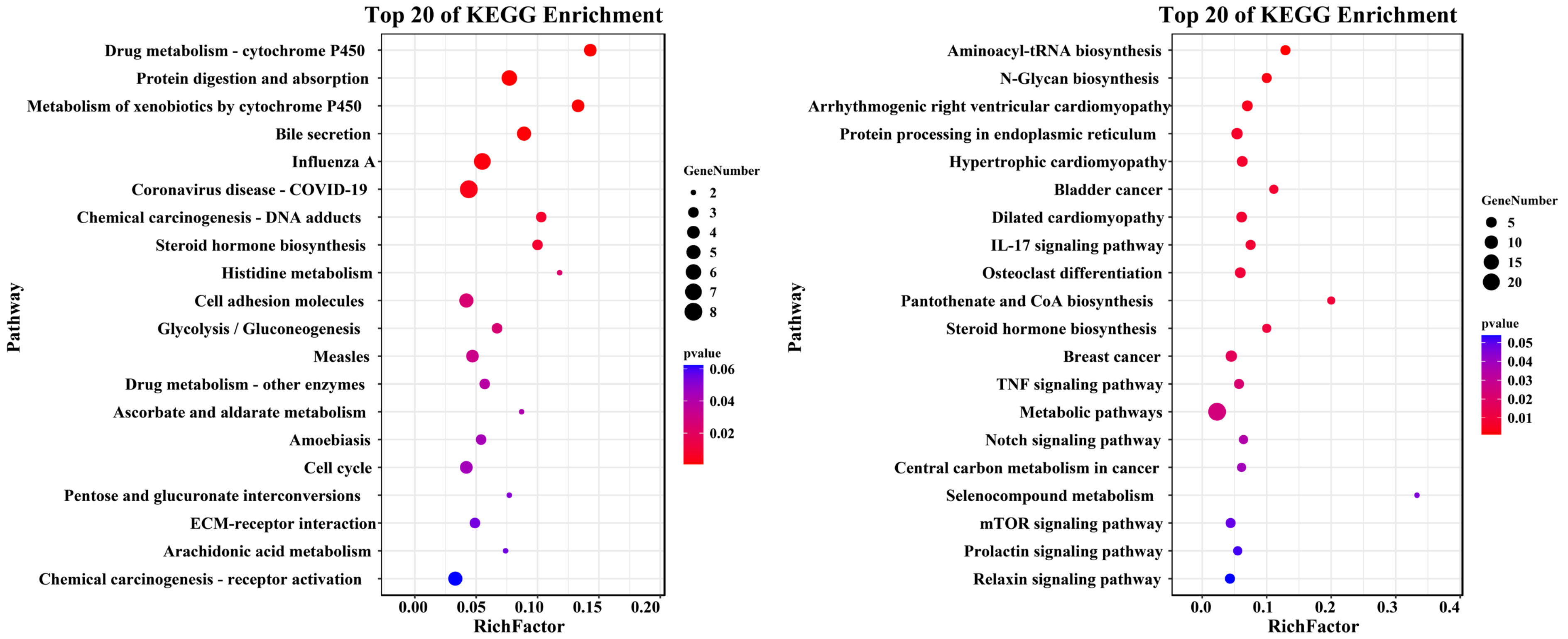

Figure S5 The KEGG enrichment result of the differentially expressed genes from the comparison of Control group vs Group 2.

a

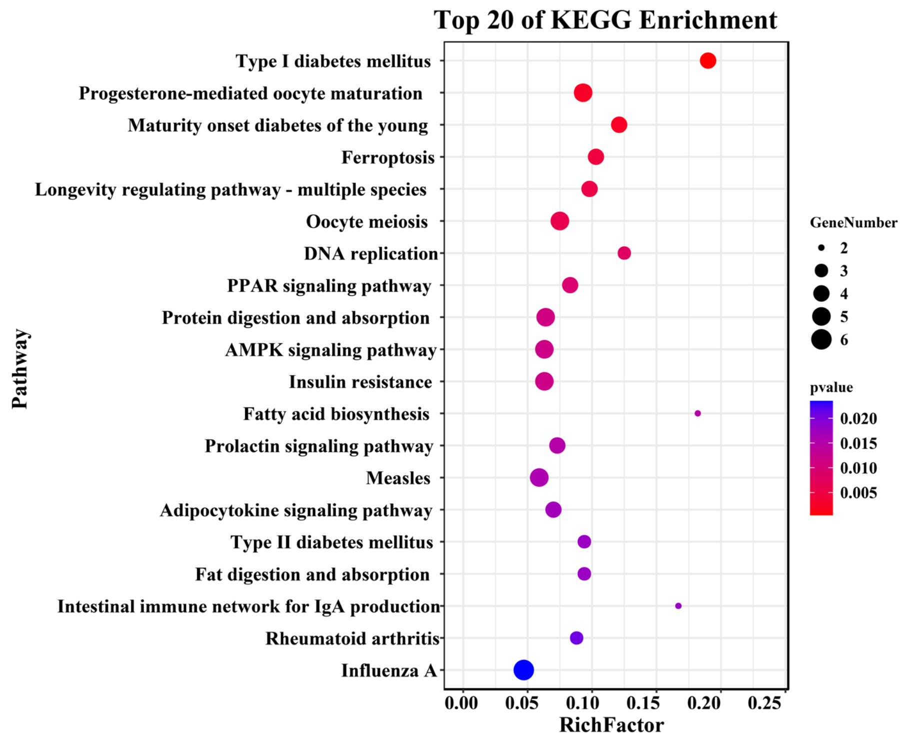

b

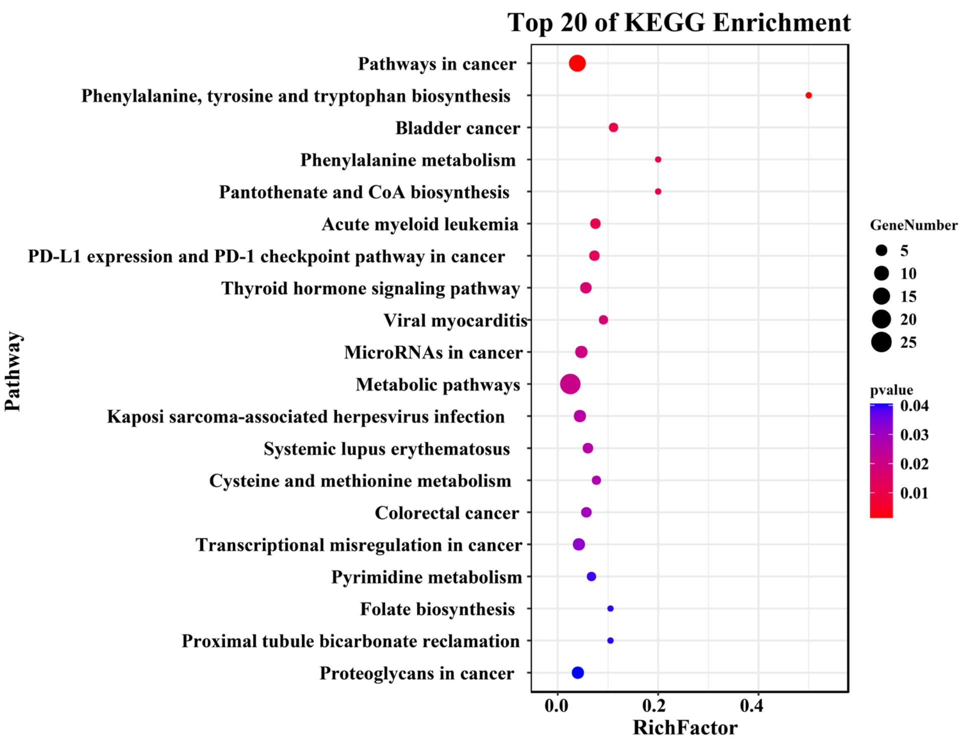

Figure S6 The KEGG enrichment result of the differentially expressed genes from the comparison of Group 1 vs Group 2.

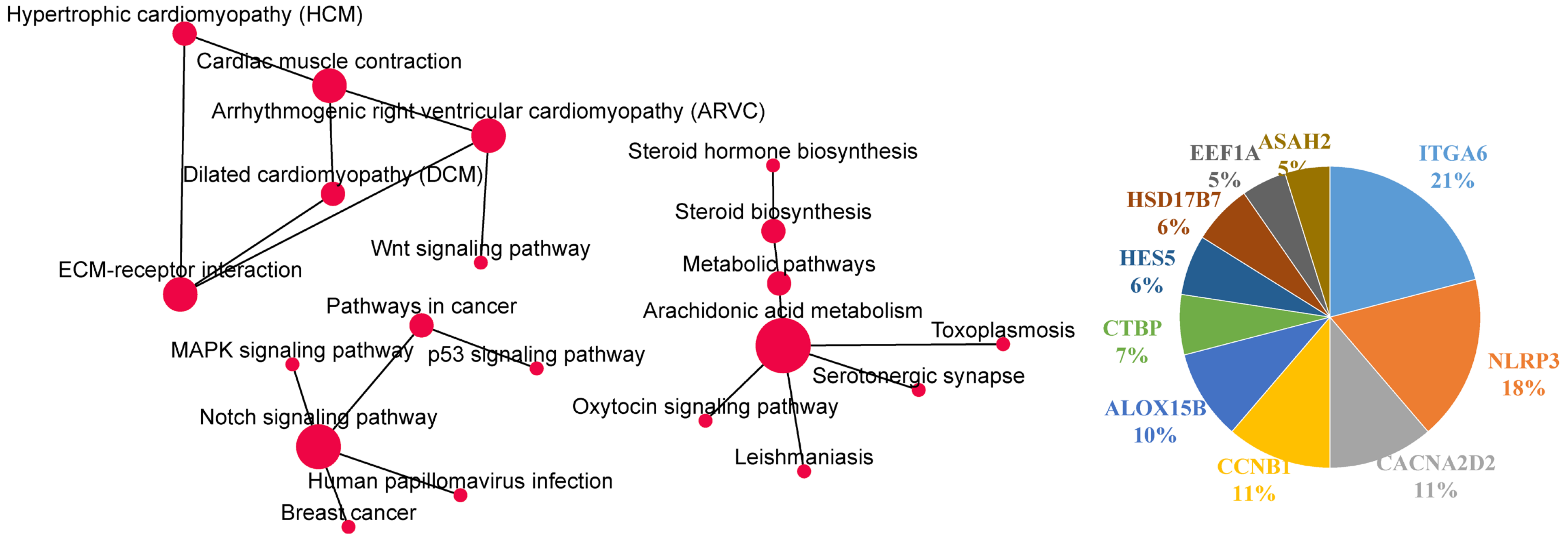

Figure S7 The KEGG enrichment result of the trend differentially expressed genes among the three groups and the major functional genes analyse

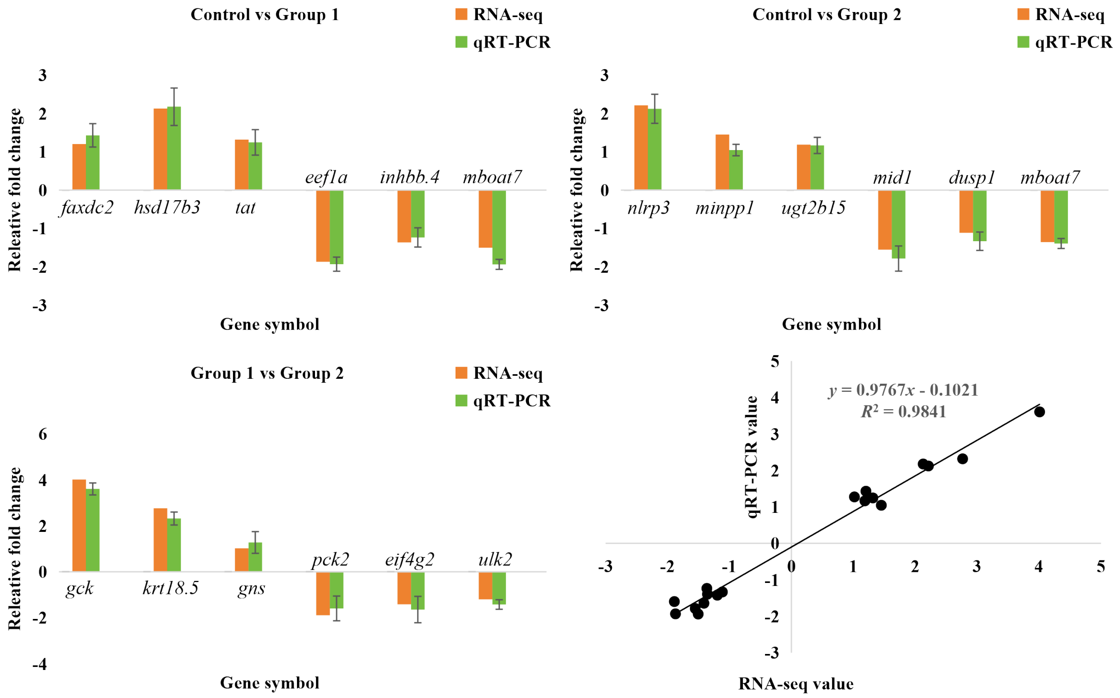

Figure S8 Results of qRT-PCR for the selected differentially expressed genes (DEGs) compared to RNA-seq.
